# Gelatin nanoparticles with tunable mechanical properties: effect of crosslinking time and loading

**DOI:** 10.3762/bjnano.13.68

**Published:** 2022-08-16

**Authors:** Agnes-Valencia Weiss, Daniel Schorr, Julia K Metz, Metin Yildirim, Saeed Ahmad Khan, Marc Schneider

**Affiliations:** 1 Department of Pharmacy, Biopharmaceutics and Pharmaceutical Technology, Saarland University, Campus C4 1, Saarbruecken, Germany; 2 Department, Drug Delivery, PharmBioTec Research and Development GmbH, Science Park 1, Saarbrücken, Germany; 3 Department of Pharmacy Services, Vocational School of Health Services, Tarsus University, Mersin, Turkey; 4 Department of Pharmacy, Kohat University of Science and Technology, 26000 Kohat, Pakistan

**Keywords:** atomic force microscopy, drug delivery, elasticity, gelatin nanoparticles, Young’s modulus

## Abstract

Tuning the elastic properties of nanoparticles intended to be used in drug delivery is of great interest. To this end, different potential formulations are developed since the particle elasticity is affecting the in vitro and in vivo performance of the nanoparticles. Here we present a method to determine the elasticity of single gelatin nanoparticles (GNPs). Furthermore, we introduce the possibility of tuning the elastic properties of gelatin nanoparticles during their preparation through crosslinking time. Young’s moduli from 5.48 to 14.26 MPa have been obtained. Additionally, the possibility to measure the elasticity of single nanoparticles revealed the influence of loading a macromolecular model drug (FITC-dextran) on the mechanical properties, which decreased with raising amounts of loaded drug. Loaded particles were significantly softer, with Young’s moduli between 1.06 and 5.79 MPa for the same crosslinking time, than the blank GNPs. In contrast to this, lysozyme as a crosslinkable macromolecule did not influence the mechanical properties. A good in vitro cell compatibility was found investigating blank GNPs and FITC-dextran-loaded GNPs in viability assays with the cancer cell line A549 and the human primary cell-derived hAELVi cell line.

## Introduction

Developing nanoparticulate drug carriers for various diseases and application routes requires establishing controllable systems, matching the needs of the respective application to achieve optimal treatment. Therefore, the characteristics of nanoparticles regarding mechanical properties, size, surface charge, surface composition, and degradation and drug release mechanisms must be considered during formulation development [1]. Except for the mechanical properties, the research activities do already cover these aspects [2]. Mechanical properties are important particle characteristics that are often overseen in the development process of drug delivery systems. Mechanical properties significantly impact the fate of particles during in vitro tests and in vivo applications. A great number of studies dealing with mechanical properties of nanoparticulate drug carriers determines the elasticity of bulk film material instead of that of the particles themselves [3]. Lack of appropriate equipment and training for measurements under physiological conditions might be the reasons. Alsharif et al. demonstrated the impact of the measurement setup for polymeric nanoparticles [4]. Young’s moduli obtained by measurements conducted on bulk materials and nanoparticles composed of PLA and PLGA vary significantly from each other. Furthermore, they showed the impact of measurements in water and at physiological relevant temperatures. Performing the measurements at body temperature or 37 °C can have a drastic effect on the resulting Young's modulus [4]. For hydrogel NPs, the influence of the experimental conditions might be even more pronounced due to the high water content [5].

The suitability of a drug carrier system is usually tested intensively in vitro using cell culture-based assays before application in in vivo studies. Depending on the particle material, the mechanical properties will be influenced, also modulating potentially the particle–cell interaction. For polymeric particles, a higher uptake for stiffer particles is explained by a lower energy needed to engulf them as demonstrated by coarse grain simulations [6]. The simulations showed a faster receptor binding for more deformable particles due to the larger surface area upon deformation. However, when it comes to membrane wrapping and internalization, the stiffer particles show significantly faster kinetics [6]. Similar results could be shown in an experimental approach by Hui et al. in a study with silica nanocapsules. Softer particles showed a greater deformation, resulting in a slower and less efficient uptake for softer particles [7]. Contrarily, nanoparticles composed of a lipid bilayer with cores of different crosslinking extent showed higher uptake of softer particles in MCF-7 cells, which was thought to be due to a melting process of particles with the cell membrane that consumed less energy than endocytosis [8].

Regarding the in vivo fate of particles after application, harder particles have a shorter blood circulation time as they are cleared faster from the bloodstream and the distribution into different tissues [3,9]. Furthermore, the particle penetration into tumor tissues could be demonstrated to be enhanced for softer particles. In this way, a higher passive drug targeting effect can be achieved, which was explained by enhanced deformability. The enhanced shape flexibility should lead to a higher extravasation rate and less effective clearance mechanisms. This combination leads to an enhanced particle accumulation in the tumor [8]. This behavior might be exploited for targeting or evading specific cell types. In this context, the cell type also plays a crucial role [7]. Overall, looking at the differences exhibited by the use of different materials for nanoparticle preparation, the favorable Young’s modulus should be evaluated during the formulation development and tuned according to the requirements of the target.

Gelatin nanoparticles (GNPs) were introduced as potential biocompatible and biodegradable drug carrier system [10–11]. This hydrogel nanoparticulate carrier system shows great potential for the delivery of macromolecules, such as proteins [12] and peptides [13], or in the field of gene delivery [14]. The surface charge of gelatin nanoparticles at physiological pH can be easily influenced by the choice of gelatin type [15]. Crosslinking of gelatin nanoparticles is still inevitable to obtain particles that are stable in aqueous environment. The choice of the crosslinker has an impact on the resulting particle characteristics, such as isoelectric point or zeta potential [10,12]. For gelatin nanoparticles, only in vitro data is presented showing the impact of mechanical properties. In the past, it was shown that the transfection efficiency of cationized gelatin nanoparticles to myeloid leukemia cells (K562) highly depends on the elasticity [16]. Furthermore, it could be shown that stiffer particles were taken up faster in the alveolar epithelial cancer cell line A549 compared to their soft counterparts. For this study, the rigidity of particles was increased by aging through a longer storage of the particles at 4 °C [17].

In the present study we established a novel experimental protocol, which enabled us to vary the mechanical properties by altering preparation parameters. Crosslinking time could be varied providing colloidally stable and soft particles. This is in contrast to the aforementioned investigations, where the particle stiffness could not be influenced by the preparation, but only increased through particle aging after a storage period. Quantitative imaging (QI™-mode) was used for the determination of the Young’s modulus of single particles. The resulting mechanical properties could be correlated with the crosslinking time and extent. Additionally, loading of the macromolecule FITC-dextran 70 kDa into the particle matrix showed no statistically significant effect on particle sizes but altered the elastic characteristics of the particles significantly resulting in softer particles in depending on the loading.

## Results and Discussion

### Size and morphology

For all examined crosslinking times between 0.5 and 3 h the size of gelatin nanoparticles ranged from 194.6 to 232.39 nm with a polydispersity index (PdI) from 0.075 to 0.115, indicating a narrow size distribution. Crosslinking for 15 min did not result in stable particles with uniform colloidal properties. The surface charge of gelatin nanoparticles is clearly pH-dependent. At a pH value of 7.5 ± 0.1, the zeta potential values ranged from −14.89 to −20.25 mV showing a slight increase for particles crosslinked for a longer time. This can be explained by the consumption of lysine groups during the crosslinking process, resulting in less positively charged functional groups.

**Figure 1:**
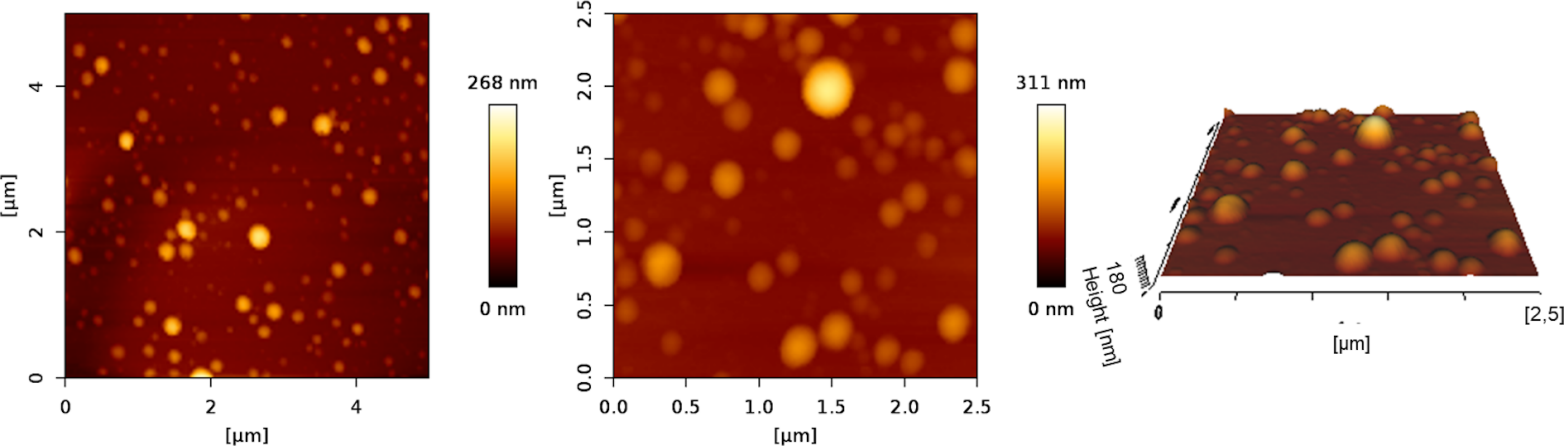
2D and 3D height images of gelatin nanoparticles crosslinked for 0.5 h. Images were taken at 37 °C in Milli-Q^®^ water running the JPK Nano Wizard 3 AFM executing the QI™-Mode.

The loading of FITC-dextran did not influence either size or size distribution. The zeta potential of FITC-dextran-loaded particles was slightly increased in comparison to the unloaded ones, which can be due to a slightly lower crosslinking rate. Sizes, PdI, and zeta potential of all formulations are listed in Table 1. Particle imaging in the QI™-mode allowed to extract the height image from the force–distance curves acquired at each pixel. Particles were well distributed on substrates showing no agglomeration and a narrow size distribution. GNPs occur with a smooth surface and are spherically shaped when measured under liquid conditions. Particles crosslinked for 0.5 h imaged under liquid conditions by AFM are shown in Figure 1. Incorporating lysozyme as a protein drug up to an initial loading of 3 mg per 20 mg gelatin resulted in particles with comparable size (242.67 ± 11.32 nm), size distribution (0.132 ± 0.04), and a negative zeta potential at neutral pH (−36.0 ± 1.18 mV). The achieved entrapment efficiency was comparable to the loading determined in a previous study in our lab [12].

**Table 1:** Physicochemical characterization of GNPs crosslinked for specific times, dextran-loaded with an initial loading of 1 mg FITC-dextran per 20 mg gelatin and blank particles. The size is displayed as mean z-average in nanometers, PdI values were calculated using the ZetaSizer Explorer software, the zeta potential at a pH of 7.5 is shown in millivolts, the crosslinking degree was determined using TNBS assay and displayed in % of free primary amine functions, and the loading of gelatin nanoparticles was obtained by fluorescence intensity measurements and is shown as micrograms FITC-dextran per milligram nanoparticles.

Crosslinking-time [h]	Size [nm]	PdI	Zeta potential [mV]	Crosslinking degree [%]	Loading [µg/mg]

0.5 Blank	212.6 ± 19.15	0.115 ± 0.017	−14.89 ± 1.78	54.61 ± 8.02	—
1 Blank	232.39 ± 17.69	0.105 ± 0.012	−16.93 ± 0.53	60.09 ± 0.76	—
2 Blank	229.43 ± 17.02	0.123 ± 0.011	−15.57 ± 0.79	73.63 ± 1.57	—
3 Blank	194.6 ± 13.55	0.093 ± 0.005	−18.59 ± 0.80	74.83 ± 0.49	—
0.5 FITC-Dext	225.4 ± 17.01	0.112 ± 0.014	−15.2 ± 0.79	49.96 ± 4.50	4.29 ± 3.33
1 FITC-Dext	221.8 ± 12.65	0.114 ± 0.008	−17.35 ± 0.85	55.19 ± 9.45	5.37 ± 2.42
2 FITC-Dext	226.35 ± 16.83	0.116 ± 0.014	−17.94 ± 0.64	63.34 ±6.32	9.31 ± 3.31
3 FITC-Dext	195.45 ± 13.08	0.075 ± 0.024	−20.25 ± 0.41	66.80 ± 8.52	18.96 ± 7.37

### Crosslinking degree and Young’s moduli

**Figure 2:**
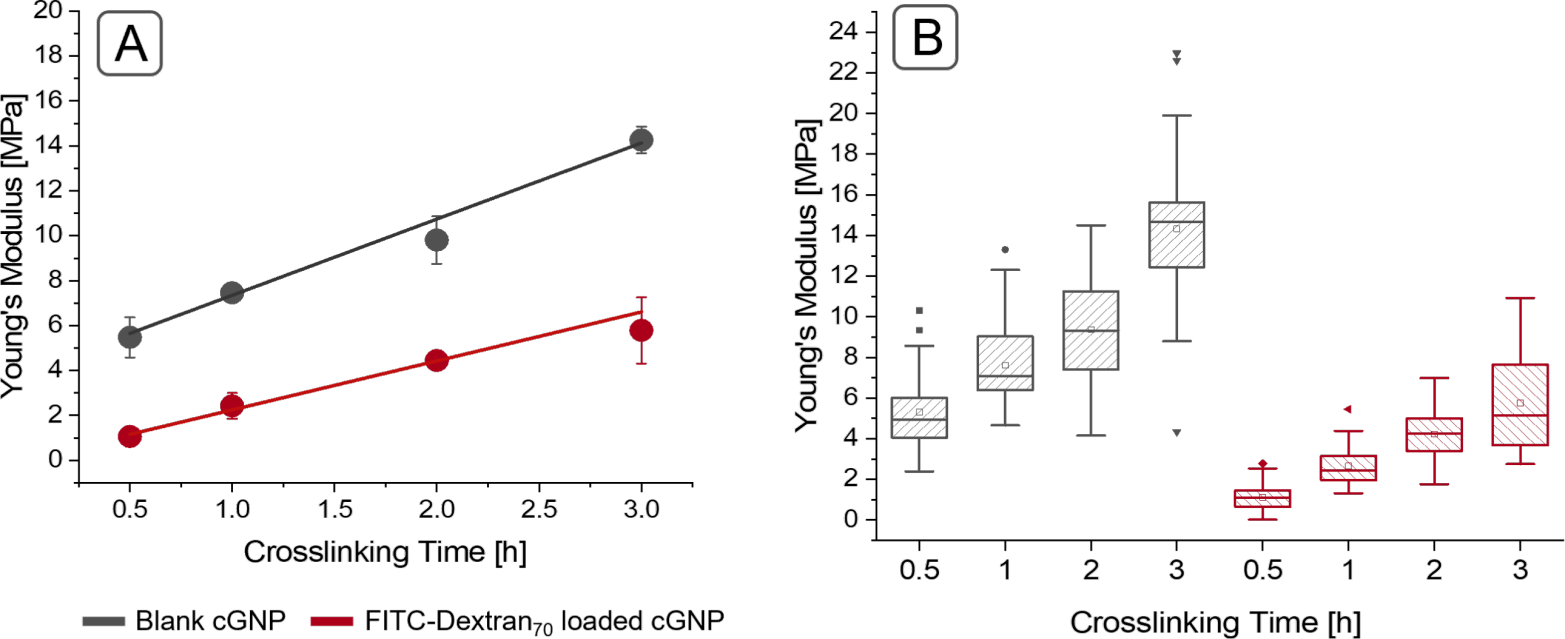
Young’s moduli of gelatin nanoparticles and crosslinking time. (A) Highlighting the linear dependency of crosslinking time and the resulting mechanical properties. Using the mean Young’s modulus of each batch to calculate the overall mean of each respective formulation. Unloaded particles in grey and particles loaded with FITC-dextran 70 kDa in red. (B) Showing the distribution of Young’s moduli by plotting boxplots with all measurement values. Looking at the Young’s moduli after the tested crosslinking times as well as comparing loaded particles with blank gelatin nanoparticles of the same crosslinking time, the difference of the resulting mechanical properties is statistically significant.

Regarding the mechanical properties of the gelatin nanoparticles, a clear dependency of Young’s moduli on the crosslinking time could be observed. For exactly controlling the crosslinking time, the crosslinking reaction was abruptly stopped by the addition of sodium metabisulfite neutralizing unbound glutaraldehyde. There is a linear correlation between crosslinking time and Young’s moduli of the particles, as evident from the regression coefficient value of 0.9869 for blank gelatin nanoparticles. Young’s moduli values ranged from 5.48 to 14.26 MPa for 30 min crosslinking and 3 h crosslinking, respectively. This data is supported and can be explained by the crosslinking degree of GNPs, which shows an increase for particles that have been crosslinked for a longer time. A glutaraldehyde incubation time of 0.5 h resulted in a crosslinking degree of 54.3%. Increasing the crosslinking time to 3 h raised the crosslinking degree to 73.8%. Approximately 70% crosslinked primary amine functionalities seem to be the maximum possible degree when crosslinking gelatin nanoparticles with glutaraldehyde. Data in Table 1 as well as previously published data by Khan et al. for 24 h of crosslinking support this conclusion [18]. The Young’s moduli of gelatin nanoparticles in dependency of the crosslinking time are displayed in Figure 2. It can be seen from the 25 to 75% area in the boxplots displayed in Figure 2B. Furthermore, it is expressed by the standard deviation of the mean Young’s moduli. The difference of the resulting Young’s moduli after different crosslinking times was tested by one-way ANOVA and was found to be statistically significant for all different crosslinking times. The absolut Young's modulus values are significantly lower than reported before [17]. This can be due to different reasons such as a batch to batch variance of gelatin as a natural product, a difference in the measurement speed during the AFM experiment and the introduction of sodium metabisulfite in the prepation process.

Comparing the crosslinking degree and the mechanical properties as functions of the time, it is obvious that the Young’s moduli are increasing even though the crosslinking degree is reaching a plateau. A similar behavior was demonstrated by Bigi et al. for bulk gelatin gels in the past [19]. They claimed that this can be explained by the lower swelling behavior of particles crosslinked with a higher glutaraldehyde concentration [19]. The lower swelling potential resulted in a denser gelatin network (reflected by the smaller hydrodynamic sizes), which consequently showed lower elasticity. This could explain the stiffer nanoparticles after 3 h of crosslinking in comparison to the particles crosslinked for 2 h, as the longer crosslinking time results in smaller hydrodynamic particle diameters indicating a decreased swelling potential resulting in a denser network structure.

### Influence of loading on the mechanical properties

The second parameter addressed in this study is the influence of FITC-dextran loading on the mechanical properties of nanoparticles. FITC-dextran was chosen as a macromolecular model drug because it has no primary amine functions, and, therefore, it cannot be crosslinked into the particle matrix by glutaraldehyde. FITC-dextran 70 kDa was loaded into nanoparticles by incorporation during precipitation and subsequent crosslinking of the nanoparticles. The fluorescence intensity was measured after enzymatic degradation of the particles using trypsin for 6 h. The results revealed a crosslinking-dependent loading, with increased FITC-dextran loading for a longer crosslinking. The initial incorporation of FITC-dextran had a significant influence on Young’s moduli. The correlation between Young’s moduli and the crosslinking time was still linear after loading, with a regression coefficient of 0.9894. Loading resulted in significantly softer particles with Young’s moduli of 1.06 MPa after 30 min of crosslinking and 5.79 MPa for particles crosslinked for 3 h, that is, the particles were between 2.2- and 5.17-fold softer than unloaded ones. The significance of the difference between the different Young’s Moduli was tested by one-way ANOVA and resulted in significant differences for all FITC-dextran-loaded samples. Comparing Young's moduli of FITC-dextran-loaded GNPs with unloaded GNPs of the same crosslinking extent the difference can be stated as statistically different, too. The softer particle characteristics can partially be explained by the lower crosslinking degree for loaded particles. However, comparing 3 h loaded GNPs with 0.5 h unloaded ones, they exhibit the same Young’s moduli but not the same crosslinking degree. Therefore, there must be an additional effect influencing the mechanical properties. The loading will most likely result in a coarser network structure as the FITC-dextran molecules will not be crosslinked but are embedded in between the gelatin chains keeping a certain distance between the gelatin polymer chains. After release, the molecules might leave pores behind, which make the particles even more deformable. A further plausible mechanism is that the gelatin network can slide along the FITC-dextran molecules and therefore shows easier deformation under external pressure, resulting in lower elastic moduli.

**Figure 3:**
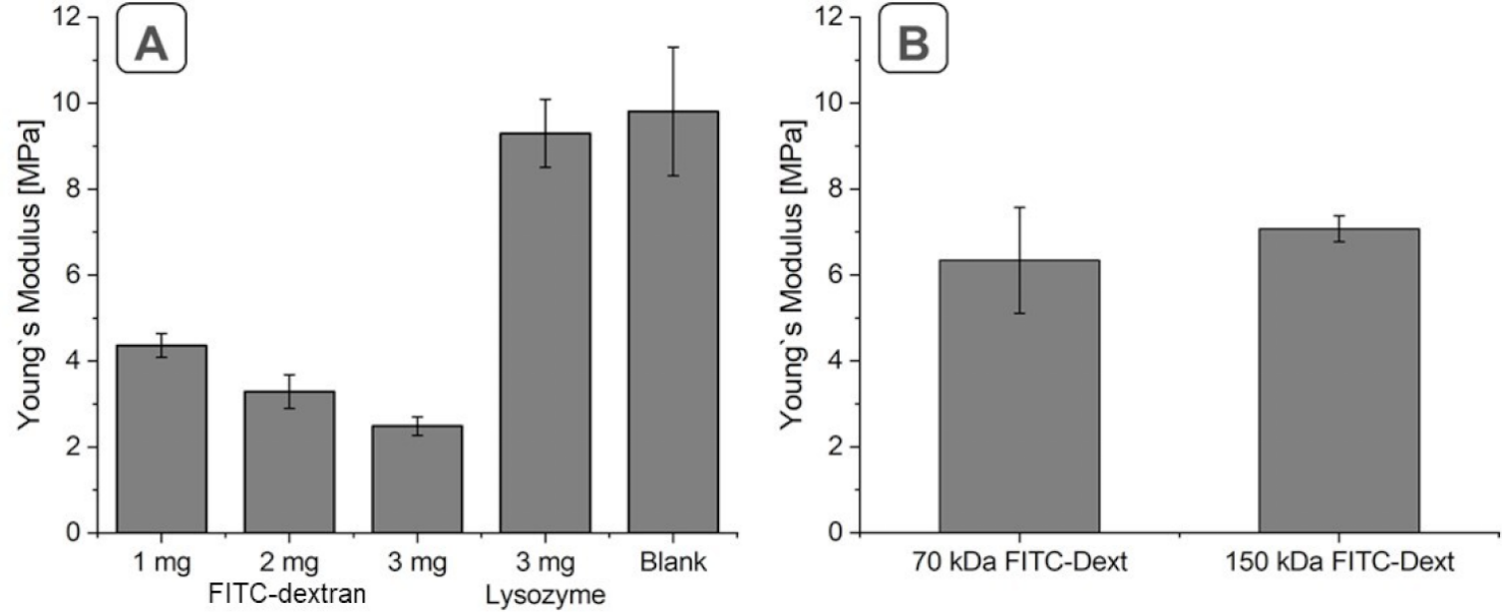
Influence of loading on the resulting particle elasticity. (A) FITC-dextran 70 kDa and lysozyme loading at different initial amounts after 2 h of crosslinking. (B) Loading of FITC-dextran with different molecular weight in the same mass concentration after 3 h of crosslinking.

Increasing the loaded amount of FITC-dextran resulted in an enhanced reduction of the elasticity. The Young’s moduli of 2 h crosslinked GNPs loaded with initial amounts of 1 mg, 2 mg, or 3 mg are depicted in Figure 3A. The resulting Young’s moduli significantly decreased from 4.36 to 2.48 MPa. Changing the molecular weight of the incorporated compound while keeping the absolute amount constant should also show an influence on the particle elasticity, due to the changed number of molecules. Comparing an initial amount of 1 mg 70 kDa FITC-dextran per 20 mg gelatin (our standard approach) with 1 mg of 150 kDA FITC-dextran did not show a significant difference in the elasticity. The results are depicted in Figure 3B. Although there was no statistically significant difference observable comparing 3 h crosslinked GNPs loaded with the two investigated different molecular weights, we could see a trend to stiffer particles with the higher molecular weight. Even though the effect is small it was expectable as the use of the FITC-dextran with higher molecular weight provided a lower number of molecules, which can contribute to particle softening. Adding a crosslinkable macromolecule to the matrix should, following our explanation, not show an effect on the elasticity of the particles as no “defects” in the network will be introduced. In line with this explanation, lysozyme-loaded GNPs exhibited a particle elasticity not significantly different from unloaded GNPs. Regarding the elasticity of 2 h crosslinked blank particles and particles loaded with 3 mg of lysozyme and crosslinked for the same time, Young’s moduli of 9.3 ± 0.79 MPa and 9.81 ± 1.50 MPa, respectively, were obtained. This can be explained by the covalent incorporation of the loaded drug into the particle matrix. Thus, the flexibility of the matrix is not influenced as the added macromolecule is also straightforwardly incorporated and co-crosslinked.

### Cell viability

**Figure 4:**
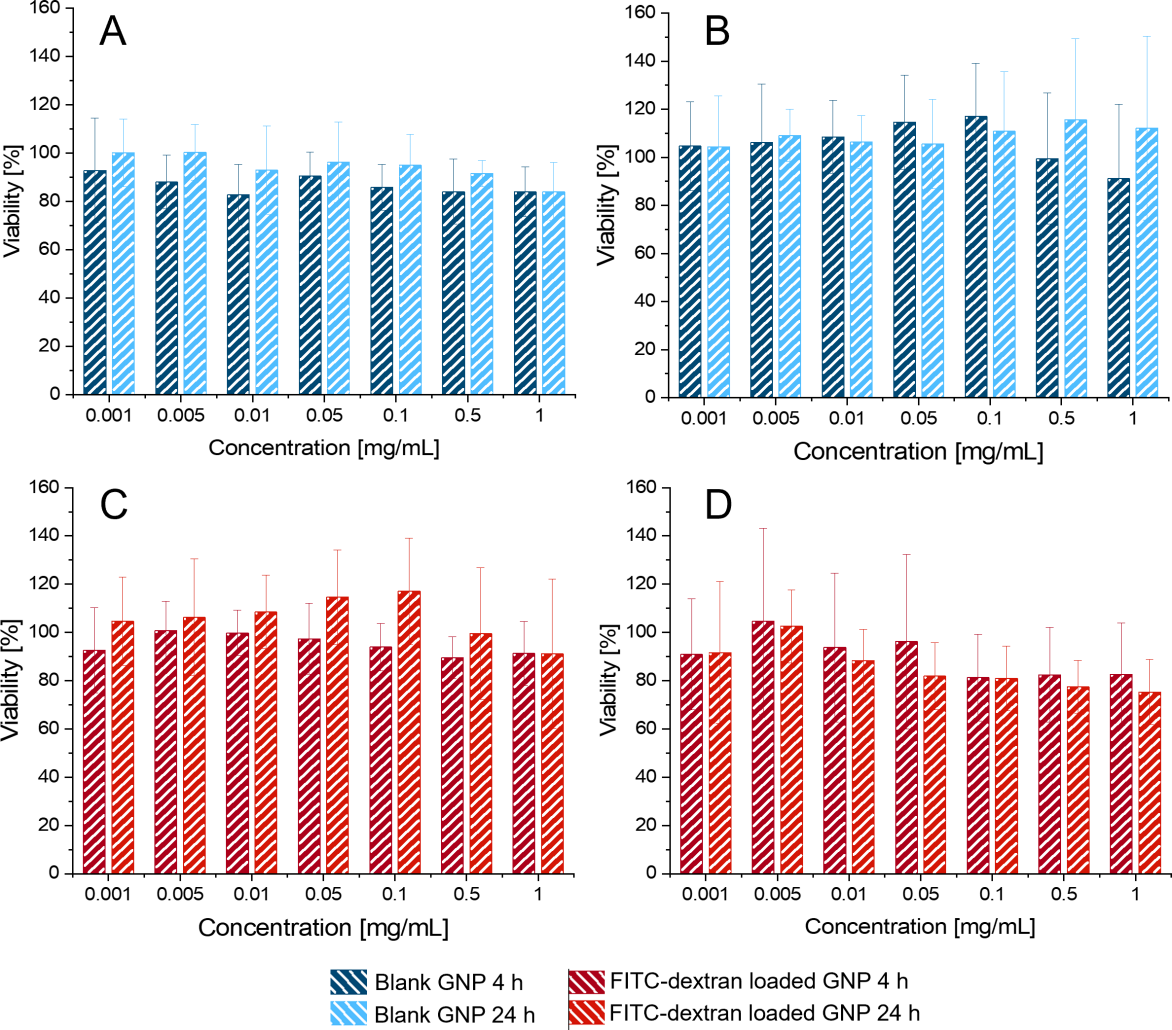
Percentage of cell viability after 4 h and 24 h exposition to 0.001 to 1 mg/mL gelatine nanoparticles measured by MTT assay. (A) A549 cells incubated with blank GNPs, (B) hAELVi incubated with blank GNPs, (C) A549 cells incubated with FITC-dextran-loaded GNPs, and (D) hAELVi incubated with FITC-dextran-loaded GNPs. No concentration- or time-dependent cell viability reduction below 80% was observable. *N* = 3 in three individual experiments.

To address potential cell viability-reducing effects of the particle formulations, these were tested on the epithelial adenocarcinoma cell line A549 and the human primary cell-derived cell line hAELVi. The cell viability was measured after incubation of blank and FITC-loaded particle formulations in concentrations ranging from 0.001 to 1 mg/mL for 4 h or 24 h with a MTT assay. No time or concentration-dependent reduction of the A549 or hAELVi cell viability below 80% relative to the controls could be detected, in neither case of exposure to blank particles or FITC-dextran-loaded particles (Figure 4).

## Conclusion

By inactivation of the crosslinking reagent, it was possible to stop the crosslinking reaction precisely. In this way, the elasticity could be adjusted by the variation of the crosslinking degree and showed a linear dependency on the crosslinking time. The obtained soft particles were colloidally stable showing sizes of 194 to 232 nm and zeta potentials between (−) 14.89 and (v) 18.59 mV. In further experiments, the loaded particles were investigated in terms of their mechanical properties. It was found that loading FITC-dextran did reduce the Young’s modulus of GNPs significantly. By increasing the loaded amount of FITC-dextran to an initial load of 2 mg FITC-dextran 70 kDa/20 mg gelatin and 3 mg FITC-dextran 70 kDa/20 mg gelatin the elasticity was further decreased. Using the same mass of a FITC-dextran with different molecular weight showed a trend towards harder particles. However, the difference was not significant. In contrast, a load that can be crosslinked into the particle matrix does not influence the overall elasticity of the particle as demonstrated for the incorporation of lysozyme. The high influence of loading on the mechanical properties demonstrates the need to determine the mechanical properties during the development of a nanoparticulate drug delivery system. For future application, the influence of different molecules loaded into GNPs and the impact of loading other polymeric particles needs to be investigated and understood. Besides this, the influence of different preparation methods is of interest.

## Experimental

### Materials

Gelatin B (average *M*_w_ 20 to 25 kDa. Bloom strength 75, Lot No.: MKBV7621V), Poloxamer 188 (Kolophor^®^ P 188), 25% glutaraldehyde solution, branched polyethylene imine (25 kDa, PEI), sodium metabisulfite, trinitrobenzenesulfonic acid 5% in methanol, sodium hydrogen carbonate, lysozyme from chicken egg white, and solvents (HPLC grade) have been obtained from Sigma-Aldrich (Steinheim, Germany). Fluorescein isothiocyanate-dextran 70 kDa (FITC-dextran) was purchased from TdB (Upsala Sweden). Pall Minimate™ tangential flow filtration capsules 300 kDa were obtained from VWR International GmbH (Darmstadt, Germany). Silicon wafers used as substrates in AFM experiments were derived from Plano GmbH (Wetzlar, Germany). MLCT Cantilevers were purchased from Bruker France Nano Surfaces (Wissembourg, France).

The human cell line A549 ACC107 [20] (DSMZ, Braunschweig, Germany) was cultivated in RPMI 1640 (Roswell Park Memorial Institute 1640, gibco™, Fisher Scientific, USA) supplemented with 10% fetal bovine serum (South American origin, PAN-Biotech, Aidenbach, Germany) and 1% v/v antibiotics (penicillin (10.000 U/mL)/streptomycin (10.000 µg/mL), Gibco™, Fisher Scientific, Waltham, MA, USA).

The human alveolar epithelial lentivirus immobilized cell line hAELVi [21], was obtained from InSCREENeX GmbH (Braunschweig, Germany) and cultured in SAGM™ (Small Airway Epithelial Cell Growth Medium BulletKit™, Lonza, Basel, Switzerland) supplemented with 1% v/v fetal calf serum (South American origin, Superior; Biochrom, Berlin, Germany) and 1% v/v antibiotics (penicillin (10.000 U/mL)/streptomycin (10.000 µg/mL), Gibco™, Fisher Scientific, Waltham, MA, USA). All plastic devices used for hAELVi culture were precoated with a 1% v/v fibronectin (Corning, NY, USA)–collagen (Sigma-Aldrich, Darmstadt, Germany) solution.

### Methods

#### Preparation of gelatin nanoparticles

The preparation was based on the nanoprecipitation method described in [17]. In brief, 20 mg gelatin was dissolved in 1 mL of deionized water at 50 °C and added to the antisolvent phase, consisting of 2.8% poloxamer 188 dissolved in a mixture of acetone and deionized water in a ratio of 15:1, with an injection rate of 0.25 mL/min using a syringe pump (Legato 200, KD Scientific Inc., Holliston, MA, USA). Immediately after completion of GNP formation, the freshly prepared particles were stabilized by adding 537 µL of 1.74% glutaraldehyde in acetone. After incubation times of 0.5, 1.0, 2.0, and 3.0 h the crosslinking reaction was stopped [22] with sodium metabisulfite, which forms a complex with glutaraldehyde, thus, terminating the crosslinking process [23]. The complex, unreacted material, and excess poloxamer 188 were removed by purification via tangential flow filtration (Minimate TFF capsule, 300 kDa, Pall Corporation, Port Washington, New York, USA). The remaining poloxamer amount was determined with a colorimetric method [24]. After six purification cycles, all free poloxamer had been removed. For the preparation of FITC-dextran-loaded GNPs, respective amounts of a 5 mg/mL FITC-dextran 70 kDa, 5 mg/mL FITC-dextran 150 kDa, or lysozyme stock solution was added to 20 mg gelatin dissolved in Milli-Q^®^ water giving a final concentration of 20 mg gelatin per milliliter containing 1, 2, or 3 mg drug per milliliter. All following preparation, purification, and characterization steps were performed as described for blank gelatin nanoparticles.

#### Crosslinking degree

Glutaraldehyde is a frequently used crosslinker in the preparation of GNPs. Each molecule of glutaraldehyde reacts with two free amine groups [25]. Translating this to gelatin, it randomly reacts with the free aliphatic amine of lysine side chains. Therefore, it is appropriate to quantify the degree of crosslinking by determining free lysine in the particles. Colorimetric determination with trinitrobenzenesulfonic acid (TNBS) is a common method [26]. Under alkaline conditions, the primary amines and TNBS react to trinitrophenyl derivatives and sulfite. The formed compound can be quantitatively measured by UV–vis spectrometry at a wavelength of λ = 349 nm related to the number of primary amines present in the equal amount of uncrosslinked gelatin. In brief, the method was as follows: 10 to 12 mg of freeze-dried particles was resuspended in 1 mL of a 4% NaHCO_3_ solution. Subsequently, an equal volume of 0.5 M TNBS in NaHCO_3_ was added. The mixture was stirred at 40 °C for 4 h. After incubation, full hydrolysis was achieved by adding 3 mL of a 6 M hydrochloride solution and autoclaving at 121 °C for 1 h. Unreacted TNBS was removed by extraction with ethyl acetate until the organic phase remained uncolored. Samples were then diluted tenfold, and the absorbance was measured at λ = 349 nm in an Infinite M200 plate reader (Tecan group, Männerdorf, Switzerland). The same procedure was followed with the same amount of pure uncrosslinked gelatin, which served as a reference.

#### Measurement of size and zeta-potential

Particle size and size distribution were measured based on dynamic light scattering using a ZetaSizer^®^ Ultra (Malvern Panalytics, Malvern, United Kingdom). 50 µL of GNP dispersion was diluted 20-fold, the pH was adjusted to 7.5, and the samples were measured in the backscatter mode. The size was evaluated as z-average, and the size distribution is displayed as polydispersity index (PdI). The zeta potential was measured by mixed measurement mode phase analysis light scattering (M3-PALS). All measurements were performed in capillary cells with a technical and an experimental triplicate.

#### Atomic force microscopy

For AFM measurements, GNPs were electrostatically fixed on positively coated silica specimens. Samples for AFM measurements were prepared according to the following protocol: Silica wafers were cleaned in an ultrasonic bath (Elmasonic B, Elma Schmidbauer GmbH, Singen, Germany) at a frequency of 37 kHz in pure ethanol for 5 min. Subsequently, wafers were dried under a flow of nitrogen and covered with an 1% aqueous solution of branched polyethyleneimine (PEI), 25 kDa, for 15 min. To remove unbound PEI, the wafers were rinsed with deionized water. Coated wafers were kept in deionized water and used on the same day. GNPs were incubated for 1 min to allow for a sufficient nanoparticle deposition without overloading the substrate surface. The supernatant was washed away with deionized water, and the samples were subsequently kept in liquid and measured on the same day.

AFM measurements were carried out with a JPK NanoWizard^®^ 3 AFM (JPK Instruments, Berlin, Germany) using the MLCT cantilever tip D (Bruker France Nano Surfaces, Wissembourg, France) with a nominal resonance frequency of 15 kHz and a spring constant of 0.03 N/m. Before each measurement, the actual sensitivity and the spring constant of the used cantilever were calibrated on a cleaned silica wafer by the thermal noise method by Hutter et al. [27] using a correction factor of 0.251. The data was acquired using the quantitative imaging mode (QI™) with image sizes of 5 × 5 µm and a resolution of 128 × 128 pixels. A loading force of 1 nN was applied and the pixel time was set to 50 ms per pixel. The setup allowed for imaging and force measurements in parallel. Thus, particles were not exposed to any forces before measuring the elastic values, reducing any measuring artefacts due to possible time-dependent elastic behavior.

#### Data processing of AFM

AFM data were processed using the JPK SPM Data Processing program (DP) version 6.1.111. Force curves were extracted from the generated files, and four force–distance curves per particle were selected from pixels representing the middle of single nanoparticles. For the values of Young’s moduli, the respective curves were treated as follows: The determined spring constant and sensitivity must be applied to calibrate the cantilever deflection. To correct the vertical offset, a baseline subtraction is applied, and the contact point is determined. The curve obtained during the AFM experiment is based on the piezo movement. The piezo movement is larger than the indentation into the nanoparticle as the cantilever bends to the opposite direction. This effect is corrected by the so-called tip–sample separation, leading to the actual indentation curve. Subsequently, the force–distance curves are fitted by the Hertz equation modified to use square-pyramidal probes according to Bilodeau [28]. Thus, Young’s moduli are obtained. Subsequently, averages of the selected curves were calculated per particle. Therefore, per batch at least 30 particles where selected. Each particle represents the mean value of four pixels located in the middle of the particle resulting in at least 120 force–distance-curves per batch. Finally, the mean value of each formulation was determined using OriginPro software.

#### Determination of FITC-dextran loading

The loading of FITC-dextran was determined by dissolving the gelatin network using enzymatic degradation. The fluorescence intensity of the resulting solution was measured [18]. Therefore, 5 mg of freeze-dried GNPs were dispersed in 5 mL PBS containing 2.5 mg trypsin at room temperature. After 6 h of incubation, samples were centrifuged. The fluorescence intensity of supernatants was measured with the spectrofluorometer Duetta™ (Horiba Europe GmbH, Oberursel, Germany) using an excitation wavelength of 493 nm and an emission wavelength of 519 nm. Concentrations were calculated using a calibration row made by different FITC-dextran concentrations in PBS.

#### Determination of lysozyme loading

The loaded amount of lysozyme was investigated using the indirect method by reverse-phase high-pressure liquid chromatography (HPLC) with a method previously reported and validated by our group [12] using an Ultimate 3000 HPLC System (Thermo Fisher Scientific, Waltham, MA, USA) with a quaternary pump, a column oven, a LichroSphere^®^ 100 RP 18 column (Merck KGaA, Darmstadt, Germany), and a UV–vis detector. The mobile phase consisted of solvent A: 10% acetonitrile, 90% MiIli-Q^®^ water and 0.1% trifluoro acetic acid and B: 90% acetonitrile, 10% Milli-Q^®^ water and 0.1% trifluoro acetic acid. The flow rate was 0.8 mL/min, and a gradient was set from 100% A to 100% B in 15 min. The concentration was determined using a calibration row by different lysozyme concentrations and the resulting AUC/min extracted from the chromatogram covering the relevant concentrations.

#### Cell viability

For determining the cell viability after particle exposure, MTT assays were performed. Therefore, 2 × 10^5^ cells/mL of A549 or hAELVi were seeded in a 96-well plate (Greiner Bio-one, Frickenhausen, Germany) in a medium volume of 200 µL. After 24 h, the cells were visualized by light microscopy (PrimoVert, Zeiss, Oberkochen, Germany) to ensure that nearly 100% confluence of the epithelial cells in the 96-well plate was reached. Before starting particle exposure, the cells were washed twice with Hanks Balanced Salt Solution (HBSS). Afterwards, the appropriate particle concentration in 200 µL HBSS was applied. After an incubation time of 4 or 24 h on a shaker with 35 rpm at 37 °C, the particle dispersion was removed, and the cells were washed once with HBSS. The MTT reagent (methylthiazolyldiphenyl tetrazolium bromide, Acros organics, USA) was added in a concentration of 0.5 mg/mL and the cells were incubated for 4 h on a shaker with 35 rpm at 37 °C protected from light. Subsequently, the absorbance was measured at 550 nm with a plate reader (Synergy 2, BioTek Instruments GmbH, Bad Friedrichshall, Germany). The resulting cell viability after substance exposure was calculated based on the absorbance measurements obtained from MTT assay by a percentual ratio to the positive control of 1% Triton X-100 (PanReac AppliChem ITW Reagents, Darmstadt, Germany) and the negative control treated with HBSS according to Equation 1.


[1] $$\text{Viability}\left[\%\right]=\frac{\left({\text{absorbance}}_{\text{particles}}-{\text{absorbance}}_{1\%\text{TritonX}-100}\right)}{{\text{absorbance}}_{\text{HBSS}}-{\text{absorbance}}_{1\%\text{TritonX}-100}}\times 100$$

#### Statistical analysis

If not stated differently, results are displayed as mean values (*n*) ± standard deviation (SD). A minimum of three independent experiments was performed for each nanoparticle formulation. Statistical significance was tested by an analysis of variance (ANOVA). It was performed as one-way ANOVA followed by Bonferroni multiple comparisons test to assess the differences between the different examined formulations. Statistical difference was assumed when *p* < 0.05. Statistical evaluations were made by OriginPro Version 2019b (OriginLab Corporation, Northampton, MA, USA).
